# Supplementary material for: Radiological correlates of vocal fold bowing as markers of Parkinson’s disease progression: A cross-sectional study utilizing dynamic laryngeal CT
Source: PLoS One. 2021 Oct 15;16(10):e0258786. doi: 10.1371/journal.pone.0258786 (PMC8519464; doi:10.1371/journal.pone.0258786)
Supplement: S1 Table — Table of summary statistics for each laryngeal measure by patient. Mean GA, IAD and IAI and their standard deviations (SD) during the vocalization period are listed. The effective minimum GA and IAD and effective maximum IAI listed refers to the median of the five lowest or highest values, whilst the inter-quartile range (IQR) given corresponds to the IQR for these five values. (DOCX) [file pone.0258786.s001.docx]

**S1 Table. Laryngeal measures during vocalization per participant**

| **ID** | **Age**  **(years)** | **Sex** | **Group** | **Duration (months)** | **UPDRS Part-III** | **GA (mm^2^)** | | | | **IAD (mm)** | | | | **IAI** | | | |
| --- | --- | --- | --- | --- | --- | --- | --- | --- | --- | --- | --- | --- | --- | --- | --- | --- | --- |
|  |  |  |  |  |  | **Mean** | **SD** | **Effective Minimum** | **IQR** | **Mean** | **SD** | **Effective Minimum** | **IQR** | **Mean** | **SD** | **Effective Maximum** | **IQR** |
| 1 | 73 | M | PD | 45 | 19 | 52.77 | 27.94 | 17.3 | 14 - 18.3 | 7.13 | 2.167 | 3.817 | 3.2 - 4.76 | 1.052 | 0.275 | 1.475 | 1.39 - 1.57 |
| 2 | 71 | M | PD | 103 | 20 | 85.43 | 59.02 | 18.62 | 14.7 - 25 | 9.22 | 2.943 | 5.488 | 5.34 - 5.53 | 1.097 | 0.634 | 2.152 | 2.05 - 2.3 |
| 3 | 67 | F | Control | NA | NA | 31.92 | 15.45 | 4.93 | 4.47 - 6.14 | 4.86 | 0.933 | 3.761 | 3.49 - 3.88 | 1.141 | 0.349 | 1.527 | 1.43 - 1.65 |
| 4 | 73 | M | PD | 39 | 16 | 13.15 | 8.29 | 4.1 | 3.9 - 5 | 3.47 | 0.592 | 2.623 | 2.54 - 2.77 | 1.015 | 0.339 | 1.563 | 1.49 - 1.67 |
| 5 | 65 | F | Control | NA | NA | 3.75 | 2.91 | 0 | 0 - 0 | 4.78 | 0.785 | 3.693 | 3.57 - 4.13 | 0.336 | 0.158 | 0.513 | 0.489 - 0.536 |
| 6 | 76 | M | PD | 41 | 12 | 36.96 | 28.23 | 13.3 | 12.3 - 14.3 | 7.01 | 1.524 | 4.821 | 4.75 - 5.02 | 0.833 | 0.239 | 1.206 | 1.07 - 1.34 |
| 7 | 65 | F | Control | NA | NA | 9.73 | 8.62 | 0 | 0 - 0 | 4.3 | 1.389 | 2.565 | 0.844 - 2.67 | 0.975 | 1.393 | 1.425 | 1.17 - 4.5 |
| 8 | 73 | F | Control | NA | NA | 8.92 | 5.09 | 2.6 | 0.9 - 3.1 | 4.98 | 0.649 | 4.171 | 3.72 - 4.25 | 0.592 | 0.252 | 0.909 | 0.806 - 1.08 |
| 9 | 68 | M | PD | 103 | 27 | 100.16 | 38.08 | 55.8 | 49.2 - 58.3 | 3.84 | 1.066 | 2.186 | 2.03 - 2.25 | 2.858 | 1.179 | 4.916 | 4.02 - 5.6 |
| 10 | 78 | F | PD | 54 | 11 | 29.5 | 24.91 | 2.4 | 1.2 - 3.7 | 7.91 | 2.526 | 4.385 | 3.88 - 4.59 | 0.661 | 0.472 | 1.175 | 1.14 - 1.28 |
| 11 | 79 | M | Control | NA | NA | 23.58 | 9.8 | 13.2 | 9.8 - 15 | 5.03 | 1.343 | 3.499 | 2.9 - 3.67 | 0.993 | 0.3 | 1.286 | 1.26 - 1.59 |
| 12 | 59 | M | PD | 51 | 25 | 20.75 | 9.81 | 10.2 | 9.3 - 10.2 | 3.64 | 1.437 | 1.417 | 1.06 - 1.6 | 1.591 | 1.308 | 3.294 | 2.52 - 3.84 |
| 13 | 61 | M | PD | 83 | 12 | 21.82 | 9.16 | 10.7 | 9.3 - 10.8 | 4.81 | 0.867 | 3.828 | 3.77 - 3.94 | 0.988 | 0.297 | 1.347 | 1.3 - 1.54 |
| 14 | 69 | M | PD | 48 | 6 | 26.73 | 8.9 | 12 | 9.6 - 15.3 | 4.62 | 0.696 | 3.587 | 3.59 - 3.69 | 1.116 | 0.252 | 1.48 | 1.37 - 1.66 |
| 15 | 74 | F | PD | 48 | 11 | 10.09 | 7.3 | 4.3 | 1.8 - 4.3 | 4.84 | 1.063 | 3.483 | 2.94 - 3.5 | 0.666 | 0.296 | 1.05 | 0.943 - 1.36 |
| 16 | 78 | F | PD | 64 | 15 | 10.23 | 5.52 | 2.8 | 0 - 2.9 | 4.14 | 0.682 | 3.266 | 3.2 - 3.34 | 0.745 | 0.301 | 1.122 | 1.1 - 1.14 |
| 17 | 63 | M | PD | 103 | 21 | 42.12 | 27 | 11.9 | 8.7 - 12.1 | 5.13 | 1.577 | 2.701 | 2.04 - 2.71 | 1.357 | 0.668 | 2.255 | 2.07 - 2.75 |
| 18 | 57 | F | Control | NA | NA | 16.5 | 7.62 | 3.6 | 3.4 - 5 | 5.21 | 0.787 | 4.33 | 4 - 4.35 | 0.761 | 0.204 | 0.995 | 0.972 - 1 |
| 19 | 67 | F | Control | NA | NA | 21.24 | 15.4 | 3.7 | 0 - 3.9 | 5.87 | 1.553 | 3.613 | 3.5 - 3.86 | 0.762 | 0.395 | 1.262 | 1.08 - 1.46 |
| 20 | 74 | M | Control | NA | NA | 13.36 | 8.48 | 2.5 | 2.4 - 2.5 | 4.91 | 0.749 | 3.578 | 3.43 - 3.74 | 0.742 | 0.343 | 1.186 | 1.14 - 1.2 |
| 21 | 81 | M | Control | NA | NA | 7.47 | 7.18 | 0 | 0 - 1.3 | 2.86 | 0.697 | 1.829 | 1.78 - 1.96 | 0.852 | 0.389 | 1.403 | 1.36 - 1.42 |
| 22 | 86 | M | Control | NA | NA | 31.97 | 6.85 | 21.8 | 21.4 - 22 | 3.82 | 0.547 | 2.977 | 2.98 - 3.03 | 1.495 | 0.229 | 1.835 | 1.81 - 1.93 |
| 23 | 75 | M | PD | 82 | 19 | 49.02 | 9.56 | 36.3 | 30.6 - 38 | 5.22 | 1.178 | 3.656 | 3.54 - 4.15 | 1.403 | 0.345 | 1.921 | 1.75 - 2.03 |
| 24 | 70 | F | Control | NA | NA | 14.57 | 9.62 | 1.4 | 1 - 1.5 | 7.87 | 3.066 | 3.89 | 3.25 - 4.29 | 0.586 | 0.4 | 1.115 | 0.969 - 1.24 |
| 25 | 81 | M | Control | NA | NA | 18.76 | 17.47 | 3.1 | 1.7 - 4.2 | 5.32 | 1.691 | 3.345 | 3.33 - 3.35 | 0.782 | 0.324 | 1.214 | 1.18 - 1.23 |
| 26 | 70 | M | Control | NA | NA | 45.5 | 31.41 | 0 | 0 - 0 | 5.03 | 0.838 | 3.761 | 3.67 - 3.78 | 1.231 | 0.707 | 2.023 | 1.97 - 2.09 |
| 27 | 61 | F | Control | NA | NA | 15.78 | 6.82 | 5.4 | 4.6 - 6.3 | 6.18 | 1.618 | 4.351 | 4.3 - 4.6 | 0.645 | 0.204 | 0.906 | 0.86 - 0.926 |
| 28 | 64 | M | PD | 32 | 8 | 23.47 | 30.83 | 0.69 | 0 - 1.24 | 4.35 | 0.82 | 3.608 | 3.25 - 3.61 | 0.926 | 0.56 | 1.456 | 1.39 - 1.56 |
| 29 | 66 | M | Control | NA | NA | 95.15 | 34.32 | 48.25 | 40.5 - 49.8 | 6.31 | 2.059 | 3.81 | 3.73 - 3.83 | 1.676 | 0.617 | 2.787 | 2.68 - 3.03 |
| 30 | 71 | M | Control | NA | NA | 124.77 | 89.29 | 39.29 | 38.3 - 42.2 | 6.54 | 2.443 | 3.479 | 3.22 - 3.52 | 1.879 | 0.944 | 3.396 | 3.1 - 4.24 |
| 31 | 65 | M | Control | NA | NA | 89.04 | 37.87 | 20.87 | 15.4 - 31.2 | 7.15 | 3.132 | 3.352 | 3.14 - 3.37 | 1.517 | 0.763 | 2.501 | 2.41 - 3.2 |
| 32 | 72 | M | Control | NA | NA | 30.17 | 36.96 | 2 | 2 - 2 | 6.5 | 1.261 | 4.278 | 3.99 - 4.45 | 0.767 | 0.497 | 1.348 | 1.23 - 1.99 |
| 33 | 82 | F | PD | 32 | 23 | 36.76 | 47.15 | 0.71 | 0.35 - 1.84 | 4.4 | 1.102 | 3.177 | 2.87 - 3.33 | 0.922 | 0.545 | 1.401 | 1.32 - 2.25 |
| 34 | 75 | M | Control | NA | NA | 107.86 | 63.62 | 35.8 | 35.8 - 41 | 7.17 | 2.895 | 4.148 | 3.83 - 4.25 | 1.815 | 1.667 | 2.902 | 2.88 - 3.65 |
| 35 | 62 | F | PD | 25 | 16 | 130.71 | 71.73 | 44.81 | 43.1 - 47.6 | 4.96 | 0.827 | 3.711 | 3.46 - 4.15 | 2.357 | 0.876 | 3.622 | 3.31 - 4.08 |
| 36 | 64 | M | PD | 120 | 35 | 61.22 | 16.53 | 41.21 | 39.1 - 42.7 | 5.82 | 3.958 | 2.353 | 1.88 - 2.45 | 1.881 | 0.958 | 3.108 | 3.04 - 3.15 |
| 37 | 64 | F | PD | 144 | 20 | 74.81 | 14.65 | 61.97 | 59.4 - 65 | 1.78 | 0.811 | 1.184 | 0.585 - 1.75 | 7.322 | 6.503 | 6.218 | 4.87 - 13.3 |
| 38 | 56 | M | PD | 240 | 34 | 73.16 | 21.32 | 52.43 | 49.1 - 53.2 | 2.95 | 0.74 | 2.218 | 1.96 - 2.22 | 3.044 | 0.922 | 3.701 | 3.69 - 3.96 |
| 39 | 63 | M | PD | 180 | 14 | 53.15 | 9.01 | 41.97 | 36.6 - 42.4 | 6.06 | 0.791 | 4.794 | 4.77 - 4.96 | 1.219 | 0.197 | 1.466 | 1.38 - 1.57 |
| 40 | 68 | M | PD | 168 | 16 | 90.74 | 30.9 | 41.64 | 41.6 - 47 | 1.52 | 0.93 | 0.351 | 0.351 - 0.761 | 9.445 | 7.695 | 21.076 | 12.2 - 30 |
| 41 | 80 | F | PD | 96 | 22 | 44 | 14.89 | 23.44 | 22.8 - 23.8 | 2.87 | 0.892 | 1.453 | 1.25 - 1.81 | 2.643 | 1.489 | 3.877 | 3.49 - 5.35 |
| 42 | 76 | M | PD | 228 | 35 | 75.66 | 12.55 | 61.55 | 54.7 - 62.6 | 1.4 | 0.856 | 0.268 | 0.19 - 0.268 | 12.936 | 14.316 | 32.304 | 26.3 - 44.1 |
| 43 | 78 | M | PD | 276 | 50 | 89.36 | 26.19 | 55.55 | 52.7 - 59.5 | 5.04 | 3.763 | 2.203 | 1.92 - 2.27 | 2.652 | 1.326 | 4.721 | 3.97 - 4.93 |
| 44 | 66 | M | PD | 228 | 19 | 64.46 | 8.07 | 54.69 | 52.5 - 56.9 | 2.45 | 0.616 | 1.719 | 1.63 - 1.74 | 3.521 | 0.876 | 4.466 | 4.45 - 4.74 |
| 45 | 69 | M | PD | 84 | 34 | 79.09 | 11.53 | 69.61 | 66 - 70.5 | 1.54 | 0.41 | 1.091 | 1.08 - 1.26 | 6.237 | 1.98 | 7.388 | 6.96 - 7.89 |
| 46 | 61 | M | PD | 144 | 15 | 70.61 | 30.23 | 23.68 | 18.6 - 47.3 | 1.8 | 0.711 | 1.155 | 0.686 - 1.22 | 5.846 | 5.672 | 7.104 | 6.48 - 7.88 |
| 47 | 66 | M | PD | 108 | 32 | 96.5 | 9.84 | 86.18 | 86 - 86.8 | 2.1 | 0.71 | 1.152 | 1.13 - 1.23 | 5.347 | 2.079 | 8.157 | 7.04 - 8.94 |
| 48 | 69 | F | PD | 60 | 38 | 55.86 | 26.28 | 22.91 | 21.1 - 34.6 | 1.6 | 1.067 | 0.57 | 0.43 - 0.711 | 6.269 | 4.21 | 10.09 | 8.83 - 11.2 |
| 49 | 84 | F | PD | 63 | 43 | 25.26 | 13.18 | 6.01 | 3.09 - 8.98 | 3.32 | 0.551 | 2.727 | 2.28 - 2.76 | 1.494 | 0.586 | 2.147 | 2.09 - 2.17 |
