# Supplementary material for: Radiological correlates of vocal fold bowing as markers of Parkinson’s disease progression: A cross-sectional study utilizing dynamic laryngeal CT
Source: PLoS One. 2021 Oct 15;16(10):e0258786. doi: 10.1371/journal.pone.0258786 (PMC8519464; doi:10.1371/journal.pone.0258786)
Supplement: S2 Table — Table listing the details of the linear mixed models used to analyse the effect of duration or severity (as graded by UPDRS part-III scores) of PD on the IAD and transformed values of the GA and IAI. (PDF) [file pone.0258786.s002.pdf]

S2 Table. Details of the linear mixed models.

	Dependent variable:					
	sqrt(GA)	IAD	log(IAI + 0.5)	sqrt(GA)	IAD	log(IAI + 0.5)
	(1)	(2)	(3)	(4)	(5)	(6)
Duration	0.016***	-0.011**	0.005***			
	(0.005)	(0.005)	(0.001)			
	t = 3.130	t = -2.100	t = 3.690			
	p = 0.005	p = 0.046	p = 0.001			
UPDRS				0.097***	-0.069**	0.031***
				(0.033)	(0.032)	(0.009)
				t = 2.920	t = -2.170	t = 3.410
				p = 0.007	p = 0.040	p = 0.003
Age	-0.067	0.057	-0.018	-0.102*	0.082	-0.029*
	(0.050)	(0.052)	(0.014)	(0.055)	(0.053)	(0.015)
	t = -1.340	t = 1.090	t = -1.260	t = -1.870	t = 1.550	t = -1.970
	p = 0.193	p = 0.284	p = 0.218	p = 0.073	p = 0.133	p = 0.059
SexM	-0.244	0.997	-0.211	0.425	0.535	0.014
	(0.816)	(0.847)	(0.230)	(0.822)	(0.795)	(0.222)
	t = -0.298	t = 1.180	t = -0.919	t = 0.517	t = 0.673	t = 0.064
	p = 0.768	p = 0.250	p = 0.367	p = 0.610	p = 0.507	p = 0.950
Constant	10.100***	0.721	1.740	11.600***	-0.289	2.230**
	(3.820)	(3.960)	(1.080)	(4.050)	(3.920)	(1.090)
	t = 2.650	t = 0.182	t = 1.620	t = 2.850	t = -0.074	t = 2.040
	p = 0.009	p = 0.856	p = 0.107	p = 0.005	p = 0.942	p = 0.042
Marginal R-squared	0.179	0.083	0.245	0.185	0.099	0.243
Conditional R-squared	0.576	0.574	0.732	0.608	0.58	0.739
Observations	962	947	913	962	947	913
Log Likelihood	-1,989.000	-1,899.000	-448.000	-1,993.000	-1,899.000	-448.000
Akaike Inf. Crit.	3,989.000	3,810.000	907.000	3,997.000	3,810.000	909.000
Bayesian Inf. Crit.	4,018.000	3,840.000	936.000	4,027.000	3,839.000	938.000
Note:	⋆ p<0.1; ⋆⋆ p<0.05; ⋆⋆⋆ p<0.01					
