# Supplementary material for: Radiological correlates of vocal fold bowing as markers of Parkinson’s disease progression: A cross-sectional study utilizing dynamic laryngeal CT
Source: PLoS One. 2021 Oct 15;16(10):e0258786. doi: 10.1371/journal.pone.0258786 (PMC8519464; doi:10.1371/journal.pone.0258786)
Supplement: Attachment — Submitted filename: Response to Reviewers.docx [file pone.0258786.s004.docx]

1^st^ September 2021

To the editor and reviewers,

Thank you all once again for your time and consideration on our research article, ‘Radiological correlates of vocal fold bowing as markers of Parkinson’s disease progression: A cross-sectional study utilizing dynamic laryngeal CT’. We are now re-submitting the manuscript with alterations which we have edited in with tracked changes.

We have acknowledged your comments (quoted in grey) with our edits included (quoted in blue) in addition to our comments.

## **Journal Requirements**

The authors apply Linear mixed model statistics (line148, 213 and following): please specify which specific model was used…

Thank you for raising this issue. We have now clarified our preferred model in the results section as follows:

Line number 222-227: “The transformed values of the *IAI* in model 3, predicting the duration (b = 0.0054, SEb = 0.0014, 95% CI [0.0024, 0.0083], t(27) = 3.69, p < .001) and model 6, predicting severity (b = 0.031, SEb = 0.0089, 95% CI [0.012, 0.049], t(27) = 3.41, p = 0.002) were the preferred models for interpretation based on having the highest marginal R-squared values (see S2 Table for model details and comparisons).”

We considered age and sex to be clinically relevant fixed factors in the model and chose to retain these in the models we reported in S2 Table. When individual factors were dropped, the AIC and BIC values did not change very much so we chose to report the most clinically relevant models described in S2 Table.

Edits were also made to the better align with APA guidelines for reporting the statistical parameters of the *IAD* models in line number 219-221 and in the abstract in line number 36-37.

We also clarified the absence of significant interactions in the model terms as follows:

Line number 229-230: “Additionally, no significant interaction was found between duration of disease or severity and participant age or sex.”

We also altered the discussion section to reflect the preference for the *IAI*:

Line number 289-291: “The preferred linear mixed models based on effect size were those which treated transformed values of the *IAI* to explain disease duration or severity.”

…and report that the necessary requirements were met – this is unfortunately not given nor provided in the text body or Table S2.

We had tested for the standard statistical assumptions. We thank you for bringing to our attention that this was not made clear in the manuscript. We have now included the following to clarify this:

Line number 216-218: “Non-linear transformations were applied to the *GA* and *IAI* to achieve linearity of the data set. This allowed all models to meet all the standard statistical assumptions.”

Also, which software was used to perform statistics?

We used the R statistics and graphing software as specified at line number 110-111. We note that we had overlooked citing this software, as well as the ‘nlme’ package used to process the linear mixed models. We have now inserted citations for both software packages and amended the reference numbers thereafter in the manuscript. We thank you for bringing this to our attention.

We also note the kind comments of reviewer #2 and #3. Once again, thank you all for your time and consideration of our manuscript.

Yours sincerely,

Professor Dominic Thyagarajan

Head of Deep Brain Stimulation Program and Group Head of Movement Disorders Research

Alfred Health and Monash University, Melbourne
